# Transcriptome-Wide Identification of Novel UV-B- and Light Modulated Flavonol Pathway Genes Controlled by VviMYBF1

**DOI:** 10.3389/fpls.2017.01084

**Published:** 2017-06-22

**Authors:** Stefan Czemmel, Janine Höll, Rodrigo Loyola, Patricio Arce-Johnson, José Antonio Alcalde, José Tomás Matus, Jochen Bogs

**Affiliations:** ^1^Quantitative Biology Center, University of TübingenTübingen, Germany; ^2^Centre for Organismal Studies HeidelbergHeidelberg, Germany; ^3^Departamento de Genética Molecular y Microbiología, Pontificia Universidad Católica de ChileSantiago, Chile; ^4^Departamento de Fruticultura y Enología, Pontificia Universidad Católica de ChileSantiago, Chile; ^5^Centre for Research in Agricultural Genomics, CSIC-IRTA-UAB-UBBarcelona, Spain; ^6^Dienstleistungszentrum Ländlicher Raum Rheinpfalz, Viticulture and Enology GroupNeustadt/W, Germany; ^7^Fachhochschule BingenBingen am Rhein, Germany

**Keywords:** flavonols, MYB, gene regulation, HY5, UV-containing light, grapevine

## Abstract

Flavonols constitute a group of flavonoids with important photoprotective roles in plants. In addition, flavonol content and composition greatly influences fruit quality. We previously demonstrated that the grapevine R2R3-MYB transcription factor (TF) VviMYBF1 promotes flavonol accumulation by inducing the expression of flavonol synthase (*VviFLS1/VviFLS4*), a key step of the initial flavonol pathway. Despite this, gene networks underlying flavonol modification in grapevine including both structural and regulatory genes remain poorly understood. In order to identify flavonol modifying genes and TFs acting downstream of VviMYBF1 a microarray-based transcriptome analysis was performed on grapevine hairy roots ectopically expressing *VviMYBF1* or a Green Fluorescent Protein as control. *VviFLS1* was induced in VviMYBF1 transgenic roots and glycosylated flavonols accumulated significantly compared with control lines. Among the differentially expressed genes, potential flavonol-modifying enzymes with predicted rhamnosyltransferase (e.g., RhaT1) or glycosyltransferase (e.g., GT3) activities were identified. In addition, important TFs of the MYB and bZIP families such as the proanthocyanidin regulator VviMYBPA1 and the UV-B light responsive HY5 homolog VviHYH were significantly altered in their expression pattern by overexpression of VviMYBF1. Co-temporal expression analysis demonstrated positive correlation of *VviMYBF1* with *VviFLS1*, *VviGT3*, and *VviRhaT1* during berry development and in fruits ripened with different light and UV-B radiation conditions at field. These results show that VviMYBF1 overexpression led to the identification of novel genes of the flavonol pathway and that the flavonol modifying machinery can be influenced by agricultural practices to optimize flavonol composition in grapes.

## Introduction

**FIGURE 1:**
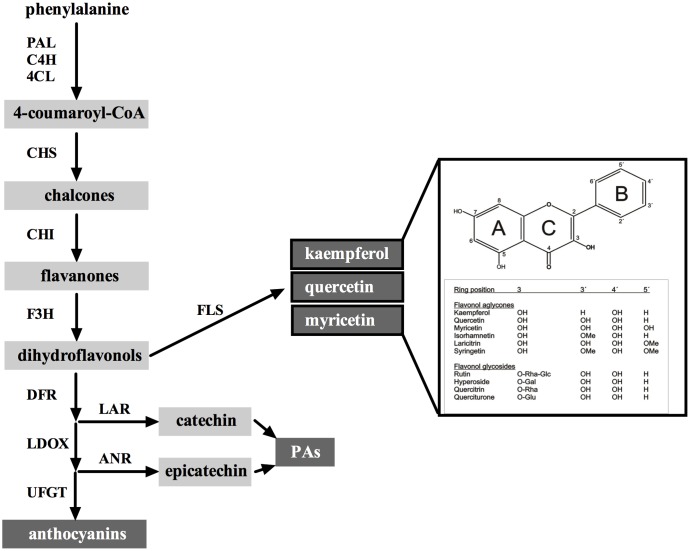
The flavonoid pathway in grapes in relation to flavonol biosynthesis. The main classes of flavonoids are indicated in dark boxes: flavonols (the three main aglycones kaempferol, quercetin, and myricetin are shown), proanthocyanidins (PAs) and anthocyanins. 4CL, 4-coumaroyl-CoA synthase; ANR, anthocyanidin reductase; C4H, cinnamate-4-hydroxylase; CHI, chalcone isomerase; CHS, chalcone synthase; DFR, dihydroflavonol 4-reductase; FLS, flavonol synthase; LAR, leucoanthocyanidin reductase; LDOX, leucoanthocyanidin dioxygenase; PAL, phenylalanine ammonia lyase; UFGT, UDP-glucose:flavonoid-3-*O*- glucosyltransferase. For simplification, flavonoids catalyzed by the enzymes PAL, C4H, DFR, and LDOX have been omitted to focus on final pathway products. A list of chemical structures from the main flavonol aglycones and some selected quercetin glycosides found in grapes is shown inside the box. Major modifications on the positions of the flavan skeleton are abbreviated as follows: Glc, glucose; Gal, galactose; Rha, rhamnose; Me, methyl group. Note that flavonols detected in grape so far are exclusively glycosylated in position 3 of the C ring.

Flavonols are the most ubiquitous flavonoids found in dietary plant-based foods (Middleton et al., 2000) and provide the second most abundant group of flavonoids in grapevine (*Vitis vinifera* L.) fruits (Georgiev et al., 2014). Flavonols largely accumulate in grape berry skins and show a remarkable facet of chemical diversity. In skins of the cultivar (cv.) ‘Shiraz’, modification of flavonols were mainly identified as 3-, 7-, and 4′-*O*-glycosylations of the basic flavonol scaffold (Downey and Rochfort, 2008; **Figure 1**). Flavonol profiles strongly depend on grapevine cultivars (Zhu et al., 2012), but in general, the main representatives in red grapes are quercetin-3-*O*-glucosides followed by myricetin, whereas quercetin and kaempferol derivatives constitute the most prominent flavonol compounds in white grapes (Mattivi et al., 2006; Castillo-Muñoz et al., 2007). The quality of red wines can be influenced by flavonols as the color of the wines is positively influenced by the copigmentation phenomenon, which is due to molecular associations between anthocyanins and flavonols or other uncolored phenolic compounds in solutions (Boulton, 2001). In recent years, it has been shown that biomedical activities of flavonols are tightly linked to their chemical diversity. The efficiency of flavonols as antioxidant compounds greatly depends on their chemical structure demonstrating a decrease in antioxidant and antibacterial capacity of flavonol glycosides compared to flavonol aglycones (Mori et al., 1987; Vinson et al., 1999; Burda and Oleszek, 2001; Montoro et al., 2005).

Although flavonols are produced as secondary metabolites, the relationship between their biological function and modification *in planta* still remains elusive. In grapevine a boost in flavonol accumulation is often observed in response to any agronomical practice that favors the exposure of grape brunches to sunlight (Teixeira et al., 2013). Consequently, flavonol biosynthesis has been extensively studied in response to its induction by UV-containing light in grapes (Price et al., 1995; Czemmel et al., 2009; Matus et al., 2009). In Arabidopsis it was shown that the *tt4* and *tt5* mutants, which are defective in the gene *CHALCONE SYNTHASE* and *CHALCONE ISOMERASE*, respectively, are sensitive to high-irradiance UV-containing light (Li et al., 1993). The authors showed that this effect was correlated with low content or even absence of kaempferol derivatives in leaves (Li et al., 1993), suggesting an important role of flavonols as UV screens *in planta*. Outside its role in UV protection, modified flavonols were found to act as endogenous flavonol inhibitors of polar auxin transport in the model plant Arabidopsis (Yin et al., 2014). Conversely the expression or activity of flavonol glycosyltransferases have been shown to be influenced by auxin levels (Santelia et al., 2008). Moreover flavonol aglycones but not flavonol 3-*O*-glycosides were able to restore pollen fertility in conditionally male-fertile petunia pollen both *in vivo* and *in vitro* (Vogt and Taylor, 1995).

Induction of flavonol biosynthesis by solar radiation can be attributed to an upregulation of flavonol biosynthetic genes leading to an increase in glycosylated flavonols. This process is negatively affected in the grape berry skin in response to light depletion (Matus et al., 2009) or UV-B filtering (Carbonell-Bejerano et al., 2014). As seen from these studies and many others, visible light and UV-B strongly affect the expression of the R2R3-MYB transcription factor (TF) *VviMYBF1* and its target, the first flavonol branch gene *FLAVONOL SYNTHASE* 1 (*VviFLS1*, also known as *FLS4* by Fujita et al., 2006; **Figure 1**), whose relation was demonstrated in grape suspension cells treated with UV-B light (Czemmel et al., 2009). Besides this well-described regulatory mechanism, only two structural genes have been identified so far involved in flavonol modification, namely *VviGT5* and *VviGT6*, which encode a UDP-glucuronic acid:flavonol-3-*O*-glucuronosyltransferase and a bifunctional UDP-glucose/UDP-galactose:flavonol-3-*O*-glucosyltransferase/galactosyltransferase, respectively (Ono et al., 2010). From these two genes, *GT5* (in addition to *MYBF1* and *FLS1*) has been directly associated with the UV-B signaling pathway in grapevine (Loyola et al., 2016).

In addition to the above-mentioned VviMYBF1, other R2R3-MYB TFs have been described as regulators of the phenylpropanoid pathway (reviewed by Czemmel et al., 2012; Matus, 2016). Similar to other plants, these TFs provide the common denominators in the regulation of structural genes of all flavonoid branches whereas co-factors encoding the basic helix–loop–helix (bHLH) domains (also referred to as MYC proteins) and conserved WD repeats (WDR) have been so far exclusively associated to the regulation of anthocyanin/PA accumulation and not to flavonol biosynthesis (Czemmel et al., 2012). The main players in grapevine are VviMYB5A and VviMYB5B proteins that are considered to be general regulators of the flavonoid biosynthetic pathway while VviMYBPA1, VviMYBPA2, and VviMYBPAR are PA biosynthesis regulators and VviMYBA1, VviMYBA2, VviMYBA6, and VviMYBA7 are specific for anthocyanin biosynthesis (Kobayashi et al., 2002; Bogs et al., 2006, 2007; Walker et al., 2007; Deluc et al., 2008; Terrier et al., 2009; Koyama et al., 2014; Matus et al., 2017).

In Arabidopsis the VviMYBF1 homologes AtMYB12, AtMYB11, and AtMYB111 regulate flavonol biosynthesis in a tissue-specific manner (Stracke et al., 2007). In both grape and Arabidopsis, flavonol related *MYBs* are regulated by the bZIP TF ELONGATED HYPOCOTYL 5 (AtHY5 and VviHY5) contributing to the establishment of UV-B tolerance in these species (Stracke et al., 2010; Loyola et al., 2016). This is in line with findings that the *hy5* mutant shows flavonol-deficient roots and downregulation of *AtMYB12* expression (Lee et al., 2007; Stracke et al., 2010).

Besides the well-established control mechanism of the initial flavonol pathway gene *VviFLS1* by VviMYBF1, it is unclear how the immense biodiversity of flavonol compounds found in grapes is achieved. In order to elucidate the regulation in biosynthesis and modification of flavonols in grapevine, *VviMYBF1* was ectopically expressed in grapevine hairy roots (HRs). Follow up experiments on these HRs allowed to identify novel structural and regulatory genes of the flavonol branch. The hypothesis that genes involved in flavonol modification were under similar transcriptional control by VviMYBF1 and environmental factors such as UV-B light was tested. Microarray analysis revealed several differentially regulated candidate genes, which are potentially involved in flavonol modification, transcriptional regulation and response to light. We selected two genes encoding for a putative flavonol rhamnosyltransferase (VviRhaT1) and a glycosyltransferase (VviGT3), which is closely related to the previously characterized flavonol modifying enzymes GT5 and GT6 (Ono et al., 2010), for downstream analysis. Expression of these candidates correlated with flavonol accumulation and *VviMYBF1* and *VviFLS1* expression in *VviMYBF1* overexpressing grapevine HRs and also in response to UV-containing light in field and greenhouse experiments. These genes are promising VviMYBF1 target structural genes that can be further investigated in relation to their impact on flavonol biosynthesis in response to common agronomical practices in the vineyard.

## Materials and Methods

### Cloning of MYBF1 Construct

The cloning of VviMYBF1 from the cultivar (cv.) ‘Shiraz’ (GenBank locus accession: FJ948477) into the vector pART7 (Gleave, 1992) to give the construct pART7MYBF1 was described previously (Czemmel et al., 2009). For generation of HRs overexpressing VviMYBF1, pART7MYBF1 was NotI digested and the insert (VviMYBF1 under control of CaMV35S) was cloned into pART27 (Gleave, 1992) to give pART27MYBF1. The binary vector pART27 contains spectinomycin resistance for bacterial selection and kanamycin resistance for selection *in planta*.

### Plant Material Collection for Developmental Series

The collection of the samples for the grapevine developmental series of *V. vinifera* cv. ‘Pinot Noir’ was described before (Höll et al., 2013). In short, sampling was performed from the start of floral initiation until harvest with early flower and later berry samples collected from a commercial vineyard during the 2007 to 2008 season in Schriesheim near Heidelberg, Germany. The berry samples derived from ∼100 berries from 20 different plants and bunches and were sampled at weekly intervals, as described previously (Downey et al., 2003). All samples were frozen in liquid nitrogen upon collection on the field and stored at -80°C until analyzed.

### Hairy Root Culture and Transformation

Before HR transformation, pART27-MYBF1 or pKGWFS7 control plasmid expressing GFP-GUS were transferred by electroporation into the *Agrobacterium rhizogenes* strain ATCC 15834, carrying the Ri plasmid pRi15834 (neither the chromosome nor the plasmid ATCC 15834 of this *A. rhizogenes* strain carry an antibiotic marker).

Transformation and induction of transgenic VviMYBF1 or GFP-GUS expressing grapevine HRs derived from leaves from *in vitro*-grown cv. ‘Chardonnay’ plants was performed exactly as described in Höll et al. (2013). After transformation and induction, transgenic HRs were routinely kept on HR) medium (Höll et al., 2013) and transferred to fresh medium after 4 weeks. HR tissues used for RNA extraction and high performance liquid chromatography (HPLC) analysis were rapidly frozen in liquid nitrogen, ground to a fine powder using a Retchmill (MM20, Retch) and stored at -80°C until further use.

### RNA Extraction and Quantitative PCR (qPCR) Analysis

Total RNA was extracted from grinded material from the developmental series of *V. vinifera* cv. ‘Pinot Noir’ as previously described in Höll et al. (2013). RNA of MYBF1 and GFP transgenic grapevine HRs was isolated following the protocol of the EURx GeneMATRIX Universal RNA purification kit (Roboklon). Prior to RT reactions, RNA samples were heated to 65°C for 10 min and immediately placed on ice to destroy secondary structures. Synthesis of cDNA was performed with SuperScript^TM^ III First-Strand Biosynthesis System (Invitrogen, catalog no. 18080-051) according to the instructions in the manual for RT using oligo dT primers. Typically, 1–2 μg of RNA were reverse transcribed in a volume of 20 μl for 1 h at 50°C followed by termination of the enzyme reaction at 85°C for 5 min. After digest of the residual RNA strands using RNase H, 30 μl of RNase free H_2_O were added. This 50 μl solution served as cDNA stock for further application and was stored at -20°C. PCR reactions were carried out for flower and berry skin samples from the cv. ‘Pinot Noir’ developmental series as well as HR samples by using 0.5 μl of 10 μM forward and 0.5 μl of 10 μM reverse primer, 5 μl cDNA (diluted 1:20), 7.5 μl of 2x ABsolute^TM^ QPCR SYBR^®^ Green Fluorescein Mix (ABgene) and 1.5 μl of H_2_O in a final volume of 15 μl (for all set of qPCR primers used in this work, see Supplementary Table 1). Thermal cycling conditions were identical for all primer pairs: 96°C for 15 min followed by 96°C for 30 s, 58°C for 30 s, and 72°C for 30 s for 35 cycles, followed by a melt cycle from 50 to 96°C. QPCR was carried out using SYBR green method for detection of double stranded PCR products on an iCyclerTM optical Module real-time cycler (Bio-Rad). *UBIQUITIN1* (previous TIGR database id: TC32075, VIT_16s0098g01190) was used for normalization of target gene levels in grapevine HRs and light experiments while the three reference genes *GAPDH* (VIT_17s0000g10430), *UBIQUITIN1* and *EF1-alpha* (VIT_06s0004g03220) were used to normalize gene expression during cv. ‘Pinot Noir’ berry development as done previously (Höll et al., 2013). The primers MYBintF and MYBintR2 were used to detect the transcript level of *VviMYBF1* in grapevine by amplification of a 214 bp PCR-fragment from the 3′ region of the gene. The primers VviFLS1 and VviFLS2 were used to detect a 154 bp amplicon of the *VviFLS1/FLS4* gene (for other primer pairs see Supplementary Table 1). The efficiency of the primers was tested in preliminary experiments with dilutions of plasmid or purified PCR products maintaining an *r*^2^ value ≥ 0.96. With all cDNAs used, the primer set gave a single PCR product, which was verified by agarose gel electrophoresis and by determination of the melt curves for the product at the end of each run.

### Flavonol Staining

Fresh cross sections from 3 to 4 weeks old HRs were transferred in a freshly prepared solution of 0.25% (w/v) diphenylboric acid 2-amino-ethyl ester (DBPA, Naturstoffreagenz A, Roth) and 0.00375% (v/v) Triton X-100. Fluorescence was visualized with either an inverted epifluorescence microscope (Leica DM IRB, Leica) or a stereomicroscope (Leica MZ FL III, Leica) using a DAPI fluorescence filter with an excitation wavelength of 340–380 nm and an emission wavelength of 425 nm. Images were captured using the Leica Image Manager (IM) 50 software and handled with Adobe Photoshop version 8.0.1 without changing the color parameters.

### Microarray Analysis

Microarray raw data generation was produced at the Centro Nacional de Biotecnologia (CNB, Madrid) by using the Grapegen GeneChip, which is an Affymetrix custom made chip containing 23,096 probe sets corresponding to ∼15,800 different annotated genes (Pontin et al., 2010) and covering ∼52% of the genome. Quality assessment of microarray data and differential expression (DE) analysis were carried out in the statistical language R (version 3.2.1) mainly using the affy (version 1.48.0) and LIMMA packages (version 3.26.9) by the Quantitative Biology Center (QBiC) in Tübingen^1^. Based on PCR analysis for the overexpressed TF *VviMYBF1* and its target *VviFLS1*, four VviMYBF1 (lines no. 32, 33, 34, and 62) and four *GFP* overexpressing lines (lines no. 77, 79, 83, and 97) out of 10 stable transformants were selected. During raw data control analysis on all eight lines (four GFP and four VviMYBF1 lines each) several sensitive measures to assess array quality such as Normalized Unscaled Standard Error (NUSE) and Relative Log Expression (RLE) confirmed low quality of array corresponding to the GFP control line 79. Since the array for line 79 was detected as an outlier, DE analysis has been performed on four VviMYBF1 and three GFP lines excluding GFP line 79. Raw data (CEL files) and meta-information (e.g., experimental design, sample names) of microarray data were deposited to NCBI’s Gene Expression Omnibus (GEO) and can be accessed under the GEO identifier GSE95532.

### Light and UV-B Radiation Treatments

Four different sunlight or UV-B radiation experiments were conducted in cv. ‘Cabernet Sauvignon’ commercial plants (either treated at field or uprooted and transferred to a greenhouse).

#### Experiment 1

Three sunlight depletion treatments were conducted at field in commercial cv. ‘Cabernet Sauvignon’ vines during the 2006–2007 ripening season, as described by Matus et al. (2009). In this study, different sunlight exposures were generated: (i) full shading of fruits by the plant’s own canopy (0% exposure), (ii) full sunlight exposure from veraison (ripening onset) onward, generated by displacement of leaves around the cluster region (100% exposure), and (iii) 25% exposure, by shading from veraison until 6 weeks after which leaves were displaced for cluster illumination.

#### Experiments 2 and 3

High (∼0.3 Wm^-2^) and Low (∼0.1 Wm^-2^) UV-B exposure treatments were applied to clusters from 9 year old potted vines in a UV-free greenhouse during 2011–2012 and 2012–2013 growing seasons, respectively, as described by Loyola et al. (2016).

#### Experiment 4

A UV-B filtering radiation treatment (here called -UV-B) was applied in cv. ‘Cabernet Sauvignon’ plants from a commercial vineyard in Maipo Valley, Chile (33° 43′ 28″ S, 70° 45′ 9.71″ W) during 2011–2012 growing season. The filtering treatment consisted in blocking solar UV-B radiation by installing a 100 μm clear polyester film at the position of grape clusters. Grape clusters from both east and west side of the rows were treated, but only grape clusters from the east side of each experimental row (exposed to sunlight during the morning until midday) were sampled. The experimental design consisted in four blocks with five plants each (biological replicates *n* = 4). Three berries per cluster (randomly sampled) and four clusters per plant were used for each sample. The treatments started at 6 weeks before veraison. Weeks -3, 0, 3, and 6 after veraison (WAV) were considered for RNA extraction and gene expression quantification while weeks -2, 1, 4, and 7 after veraison were used for HPLC analysis. A total of 60 berries were sampled for each biological replicate per condition at each sampling date. Berries were immediately peeled and deseeded. Berry skins were frozen in liquid nitrogen and stored at -80°C until RNA was extracted. Total flavonols were measured as quercetin equivalents and environmental parameters such as solar UV-B irradiance, temperature, solar radiation at cluster level and total solar radiation were measured around veraison from 08.00 to 19.45 h in the east side of the row.

### Separation of Hairy Root and Berry Skin Flavonols by HPLC

High performance liquid chromatography analysis was performed on methanolic extracts of HR lines using a reverse phase HPLC (Kontron Instruments 322 pump system/360 autosampler/335 HPLC detector; Kontron) with a Symmetry C18 column (3,5 μm, 4,6 mm × 150 mm, catalog no. WAT200632, Waters) protected by a guard column (Czemmel et al., 2009). Liquid nitrogen-frozen and subsequently homogenized sample aliquots of 20–50 mg were used to extract flavonoids by adding 200 μl of 50% (v/v) methanol (HPLC grade) in H_2_O. Samples were then sonificated for 20 min in an ice water bath and centrifuged for 10 min at 13000 rpm. 200 μl of the clear supernatant were loaded for HPLC analysis. Separation was carried out with a binary gradient of solvent A [10% formic acid (v/v) in H_2_O] to solvent B [100% (v/v) methanol, HPLC grade]. The gradient conditions were 0 min, 17% solvent B; 15 min, 35% solvent B; 40 min, 37% solvent B; 42 min, 100% solvent B; 50 min, 100% solvent B; 51 min, 17% solvent B; 58 min, 17% solvent B. The column was maintained at 40°C and the flow rate was 1 mL/min. Data acquisition and processing was performed by Kroma System 2000 software (Kontron). Data are presented as HPLC chromatogram peak areas over time and expressed as milli-absorbance units (mAU) at 520 nm for anthocyanins and at 353 nm for flavonols. Concentrations were calculated from calibration curves prepared from commercial standards (Phytolab) and expressed as quercetin-3-*O*-glucoside equivalents for flavonols and malvidin-3-*O*-glucoside equivalents for anthocyanins. Acidic hydrolysis was performed to identify the compounds as flavonols by cleaving the glycosyl group for a shift to the aglycone form by the addition of a 3 N HCL solution, 50% (v/v) methanol in water and incubation for 3 h at 95°C. All HPLC separation experiments were performed in three independent extractions of from the same biological material.

High performance liquid chromatography quantification of flavonols in berry skins from light experiments was different to flavonol measurements from HRs and berry skin and flower samples from the developmental series of cv. ‘Pinot Noir.’ For light experiments, flavonol measurements were conducted as described in Matus et al. (2009).

### Statistics

For statistical evaluation of microarray data, the LIMMA package was used (Ritchie et al., 2015). First, its function lmFit() fitted linear models on the quality controlled and RMA normalized expression values of each gene across the seven samples with the factor genotype (with the two levels MYBF1 and GFP-GUS). Then the functions eBayes() and topTable() with the argument adjust.method = “BH” were used to compute moderated *t-*statistics and extract a table of the top-ranked genes from a linear model fit with *p*-values corrected for multiple testing by applying the false discovery rate (FDR) from Benjamini and Hochberg (1995), respectively. Genes were considered differentially expressed (DE) with a FDR corrected *p* < = 0.05. No log fold change (logFC) cutoff was applied to assess DE of a gene.

To statistically assess differences in expression of genes and flavonol accumulation between VviMYBF1 and GFP control HR lines, an unpaired two-tailed *t*-test was performed with assumption of equal standard deviation (SD) between both sample groups. Results were considered statistically different at *p* < 0.05.

For each of the four UV/light experiments, differential gene expression was assessed using a two-way analysis of variance (ANOVA) with a model including main effects for the two factors time (in WAV) and treatment (+UV-B, -UV-B) and an interaction term between those two factors. To analyze at what time points the treatment effects differed, a *post hoc* analysis was performed on the model using Tukey’s test. Genes were considered statistically DE at this time point at *p* < 0.05.

Note that for accumulations of individual flavonol in response to light no statistical test was performed.

### Accession Numbers

Microarray data were submitted in the Gene Expression Omnibus (GEO) under accession number GSE95532.

## Results

### Selection of Hairy Roots for Transcriptome Analysis

**FIGURE 2:**
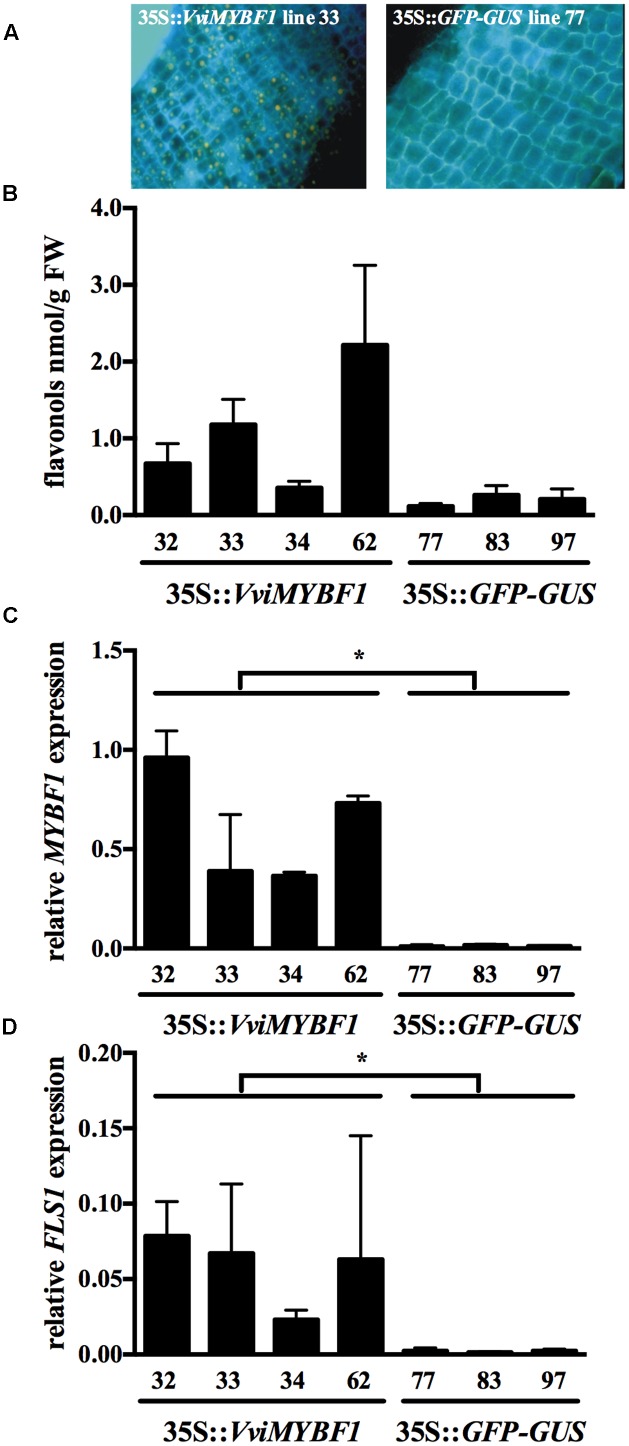
Selection of transgenic hairy root (HR) lines. Lines for microarray analysis were selected under the following criteria: flavonol accumulation as visualized by fluorescence microscopy on *in situ* diphenylboric acid-2- aminoethylester (DPBA) stained root cross sections **(A)**, flavonol content as shown by high performance liquid chromatography (HPLC) analysis **(B)** and gene expression analysis to verify transcript levels of the *VviMYBF1* transgene and its well-characterized target *VviFLS1* **(C,D)**. DBPA staining analysis shown in **(A)** was conducted with sections of the VviMYBF1 line 33 (left) and control line 77 (right). HPLC results in **(B)** are the mean values of three independent extractions from the same biological material. Gene expression analysis in **(C,D)** is shown relative to the expression of *UBIQUITIN1* gene (previous TIGR database id: TC32075, *VIT_16s0098g01190*) with standard deviations derived from three qPCR runs with three technical replicates each. Stars indicate significant differences (*p* < 0.05) between MYBF1 and control HRs based on *t*-test.

Previous studies indicate that the grapevine HR system provides a suitable tool to identify novel target genes regulated by an ectopically expressed MYB TF (Cutanda-Perez et al., 2009; Terrier et al., 2009; Khater et al., 2012; Höll et al., 2013). Transgenic HRs were generated by infiltration of *V. vinifera* cv. ‘Chardonnay’ leaves with *A. rhizogenes* carrying either VviMYBF1 or GFP (control) cDNA. Presence of *VviMYBF1* transgene was verified by PCR, whereas control lines expressing GFP were selected by PCR and fluorescence microscopy. Four VviMYBF1 (lines no. 32, 33, 34, and 62) and four *GFP* overexpressing lines (lines no. 77, 79, 83, and 97) out of 10 stable transformants were analyzed for transcript amount of *VviMYBF1* and its known target *VviFLS1* (Czemmel et al., 2009). During microarray quality control analysis, line no. 79 was detected as an outlier (for details, see Materials and Methods) and downstream analysis has been performed on four VviMYBF1 and three GFP lines excluding GFP line 79. Additionally, HR flavonols were stained *in vivo* with diphenylboric acid-2-aminoethylester (DPBA, **Figure 2A**) and quantified using HPLC (**Figure 2B**). By using fluorescence microscopy, we observed orange like bodies (indicative of the accumulation of quercetin-derivatives) exclusively in VviMYBF1 overexpressing HRs (**Figure 2A**). These bodies were prominent in epidermal cell layers while endodermal cells and parenchymatic cells of the cortex accumulate only trace amounts or did not show any orange staining indicative for flavonol accumulation. Flavonols, mainly quercetin 3-glucoside, quercetin 3-galactoside and a so far unknown flavonol derivative accumulated significantly higher in *VviMYBF1* overexpressing lines as compared to GFP control lines when measured quantitatively using HPLC (**Figure 2B**). To show that MYBF1 induces only the flavonol branch of the flavonoid pathway anthocyanins were measured in the HR lines. Neither in the *VviMYBF1* nor in the *GFP* overexpressing lines anthocyanins were detected.

With the aim to decipher the mechanisms that underlie the phenotypic changes observed in HRs ectopically expressing *VviMYBF1*, qPCR analysis was deployed. Expression of *VviMYBF1* in selected *VviMYBF1* lines exceeded significantly its expression compared to *GFP* control lines (**Figure 2C**), which resulted in a pronounced induction of its target, *VviFLS1* (**Figure 2D**). Results from this homologous expression experiment clearly support the role of VviMYBF1 in regulation of flavonol biosynthesis by induction of *VviFLS1* transcription. These results showed that selected VviMYBF1 and GFP HR lines were suitable candidates for a differential transcriptome analysis approach to identify novel target genes of VviMYBF1 involved in flavonol biosynthesis and its regulation.

### Transcriptome Analysis to Identify VviMYBF1 Target Genes

**FIGURE 3:**
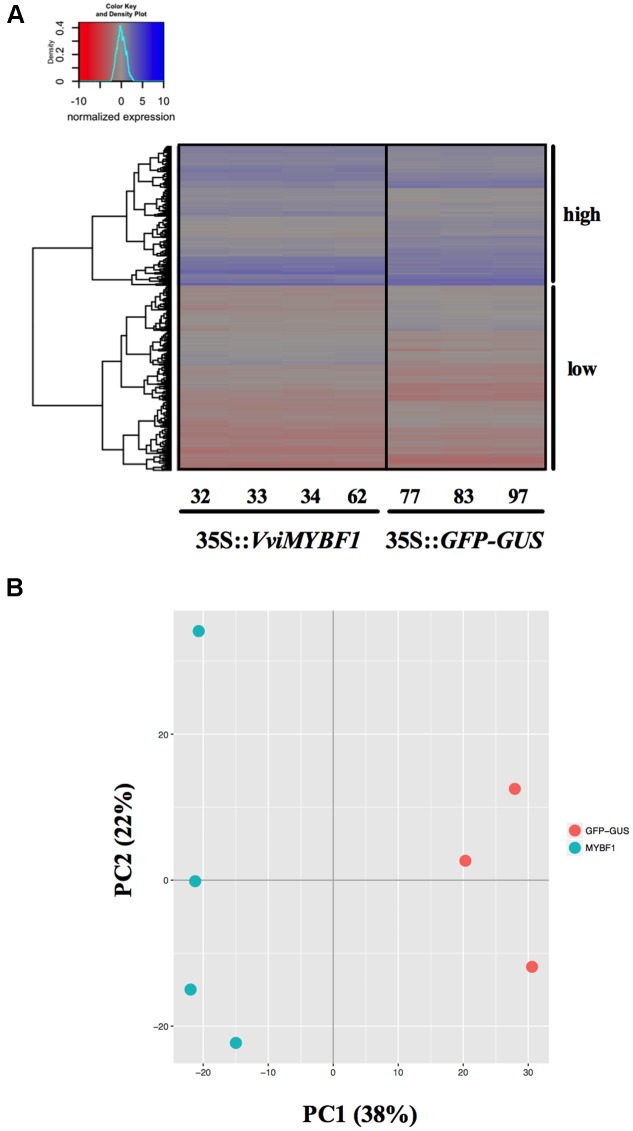
Microarray analysis. **(A)** Changes in expression of the differentially expressed (DE) probesets presented as a heat map. The dendrogram on the left in **(A)** illustrates the clustering using euclidean distance of the DE genes into two blocks based on high (blue) and low (red) expression. These two blocks are highlighted on the right. The distribution of the quantile normalized expression values is shown as color key on the topleft. Note that these values were also scaled to have mean zero and standard deviation one. Sample names are indicated on the x-axis with MYBF1 samples on the left and control samples on the right. **(B)** Principal component analysis (PCA) of gene expression in hairy roots expressing MYBF1 (blue) or GFP control (red). The plot shows the first two principal components (PC1 and PC2) that account for 38% and 22% of the total variation of the data.

To identify novel target genes of VviMYBF1, total RNA was isolated from the four VviMYBF1 and the three GFP overexpressing HR lines to perform a Microarray-based transcriptome analysis. Principal component analysis (PCA) clearly separated both, VviMYBF1 and GFP HR lines in distinct groups (**Figure 3B**) indicating differences between underlying gene expression pattern in VviMYBF1 and GFP control groups which is also visible using a heatmap (**Figure 3A**). To test the *a priori* hypothesis that genes will have significantly different mean expression values between *VviMYBF1* and *GFP* lines, pairwise comparison between the two groups VviMYBF1 and GFP was performed and moderated *t*-statistics and associated *p*-values were generated, which were further adjusted for multiple testing using Benjamini and Hochberg’s method to control the FDR (Benjamini and Hochberg, 1995). In total, 548 probesets corresponding to 484 differentially expressed genes (DEGs; that is because several oligonucleotides hybridize to the same gene) were DE based on an adjusted *p*-value smaller than 0.05 (**Table 1** and Supplementary Table 1). These 548 probesets represent ∼2% of the probes on the chip (23,096 probesets in total). 279 probesets corresponding to 230 genes have been called positive DE, while the other 269 probes, corresponding to 254 genes, show significant negative regulation in VviMYBF1 compared to GFP HR lines. Amongst the DEGs, three candidate genes were found which could modify flavonol scaffold structures (**Table 1**): a putative UDP-sugar flavonoid/flavonol glycosyltransferase [VIT_11s0052g01580, *VviGT3* (Ono et al., 2010)], a putative UDP-rhamnose:rhamnosyltransferase (VIT_00s0218g00170, *VviRhaT1*) and a putative flavonol modifying sulfotransferase (VIT_17s0000g04930, *VviST1*). An investigation of the positive DEGs for TFs revealed *VviHYH* (VIT_05s0020g01090) and *VviMYB4A* (VIT_03s0038g02310), which showed 1.5 and a 1.6 fold inductions in gene expression in VviMYBF1 versus control HRs, respectively (Supplementary Table 1). Amongst the pool of significantly downregulated transcripts several genes of the PA biosynthetic pathway were identified: the PA regulatory TF gene *VviMYBPA1* (VIT_15s0046g00170) and genes under genetic control of VviMYBPA1 including two chalcone synthase isoforms (*VviCHS3* and *VviCHS1*, VIT_05s0136g00260, VIT_14s0068g00930) and anthocyanidin reductase (*VviANR*, VIT_00s0361g00040). Microarrays also indicate that transcript abundance of a yet uncharacterized *FLAVONOL SYNTHASE* isoform, *VviFLS5* [(VIT_18s0001g03430 (Fujita et al., 2006)] was negatively affected by overexpression of VviMYBF1 (**Table 1**).

**Table 1:** Table of top differentially regulated probesets related to flavonoid biosynthesis.

Probeset	logFC	AveExpr	adj.p.val	Grapegen.unique.ID	short.Annotation
VVTU16103_at	2.68	5.76	0.02279	VIT_18s0001g03470	FLS1/FLS4
VVTU21895_at	2.05	5.54	0.01882	VIT_11s0052g01580	GT3
VVTU7774_at	1.48	8.63	0.02027	VIT_00s0218g00170	RhaT1
VVTU14806_at	1.14	9.45	0.01539	VIT_17s0000g04930	ST1
VVTU5559_at	1.57	5.89	0.00076	VIT_05s0020g01090	HYH
VVTU27986_s_at	1.63	9.19	0.00189	VIT_03s0038g02310	MYB4A
VVTU3046_s_at	-1.76	8.97	0.00137	VIT_15s0046g00170	MYBPA1
VVTU21956_s_at	-1.22	7.56	0.00855	VIT_05s0136g00260	CHS3
VVTU39820_s_at	-1.12	9.23	0.01643	VIT_14s0068g00930	CHS1
VVTU13083_at	-1.19	9.53	0.03409	VIT_00s0361g00040	ANR
VVTU2456_s_at	-1.06	7.62	0.00675	VIT_18s0001g03430	FLS5


*The probesets correspond to significantly upregulated and downregulated genes that passed the filter criteria of a multiple adjusted *p*-value of 0.05. Note that **Table 1** is sorted from highest to lowest log fold change (logFC). The respective probesets correspond to the 12Xv1 genome accession. Note that the microarray chip used here does not contain a probe corresponding to the expressed transgene *VviMYBF1*. The probeset VVTU16103 for the known VviMYBF1 target gene *VviFLS1/VviFLS4* was therefore used as a positive control for DE analysis.*

**FIGURE 4:**
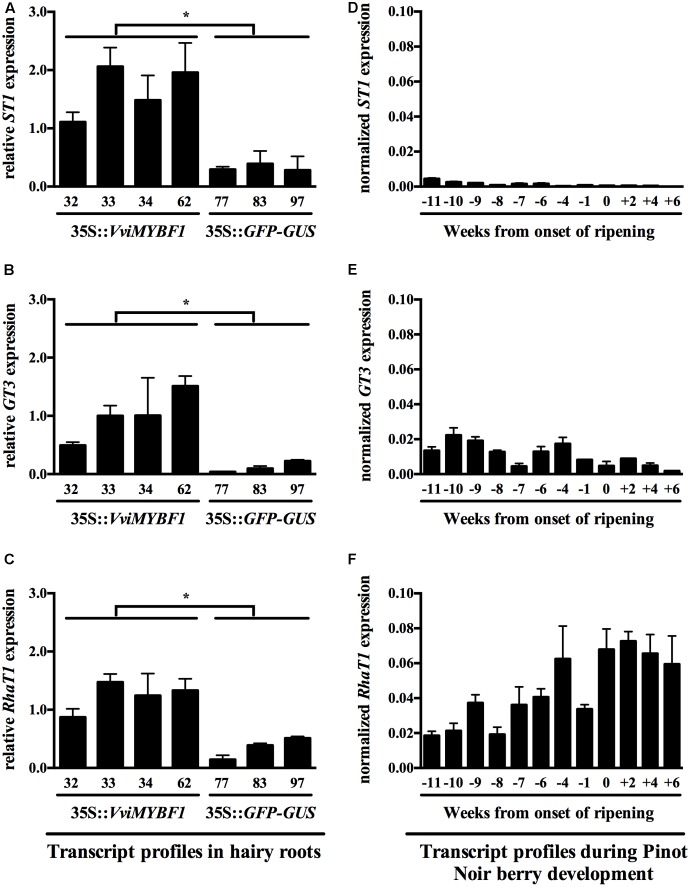
Transcript profiles of the three candidate VviMYBF1 target genes *VviRhaT1*, *VviST1*, and *VviGT3* in HRs and during berry development. Gene expression analysis in HRs **(A–C)** is shown relative to *UBIQUITIN1* expression whereas three reference genes were used to normalize expression during berry development (see Materials and Methods, **D–F)**. Data points in **(D–F)** are given as weeks from the onset of ripening (time point 0). Note that from 6 weeks before veraison (-6), berry skin has been separated from seeds. Flowering occurred 8 weeks before veraison. Each sample of the developmental series from –6 on corresponds to a pool of >100 berries collected from ∼20 plants, as described in Höll et al. (2013). Values and standard deviations are derived from two PCR runs with triplicate PCR reactions each. Stars in **(A–C)** indicate significant differences (*p* < 0.05) between MYBF1 and control HRs based on *t*-test. No statistical analysis was performed for data in **(D–F)**.

With the aim to validate the microarray experiment, expression of the three candidates flavonol-modifying genes *VviST1*, *VviRhaT1*, and *VviGT3* and the PA regulator *VviMYBPA1* were analyzed by qPCR (for primers used see Supplementary Table 1). Quantitative analysis revealed that *VviGT3*, *VviST1* and *VviRhaT1* were indeed induced in VviMYBF1 lines compared to GFP controls (**Figures 4A–C**), whereas transcript abundance of *VviMYBPA1* was negatively influenced by ectopic expression of the flavonol regulator VviMYBF1 (Supplementary Figure 1). Expression of the previously characterized flavonol modifying genes *VviGT5* and *VviGT6* were not checked in the HR samples, as probesets representing these genes were not present on the microarray chip design used here.

### Candidate Gene Expression during Grape Berry Development

For correlative analysis of gene expression during grape berry ripening, the same *V. vinifera* cv. ‘Pinot Noir’ developmental series was used, in which gene expression related to stilbene biosynthesis was measured previously (Höll et al., 2013). Expression pattern correlation between *VviMYBF1* and *VviFLS1* transcripts was highest at eleven-*to*-ten weeks prior to beginning of berry ripening (veraison), correlating with previous results on flavonol accumulation and *VviFLS1* and *VviMYBF1* expression pattern (Downey et al., 2003; Czemmel et al., 2009). Monitoring expression pattern of the putative VviMYBF1 target genes in developing berries showed that *VviST1* and *VviGT3* are low expressed during berry ripening (**Figures 4D,E**) though showing correlation with *VviFLS1* and *VviMYBF1* expressions at some early developmental stages (Downey et al., 2003; Czemmel et al., 2009). There is correlation of *VviRhaT1* expression with flavonol accumulation throughout all stages of development as *VviRhaT1* transcript amounts gradually increase from early to late stages of development (**Figure 4F**), which is similar to flavonol accumulation in grapevine (Downey et al., 2003).

### Candidate Gene Expression and Flavonol Analysis under Changes in UV-Containing Light Exposure

**FIGURE 5:**
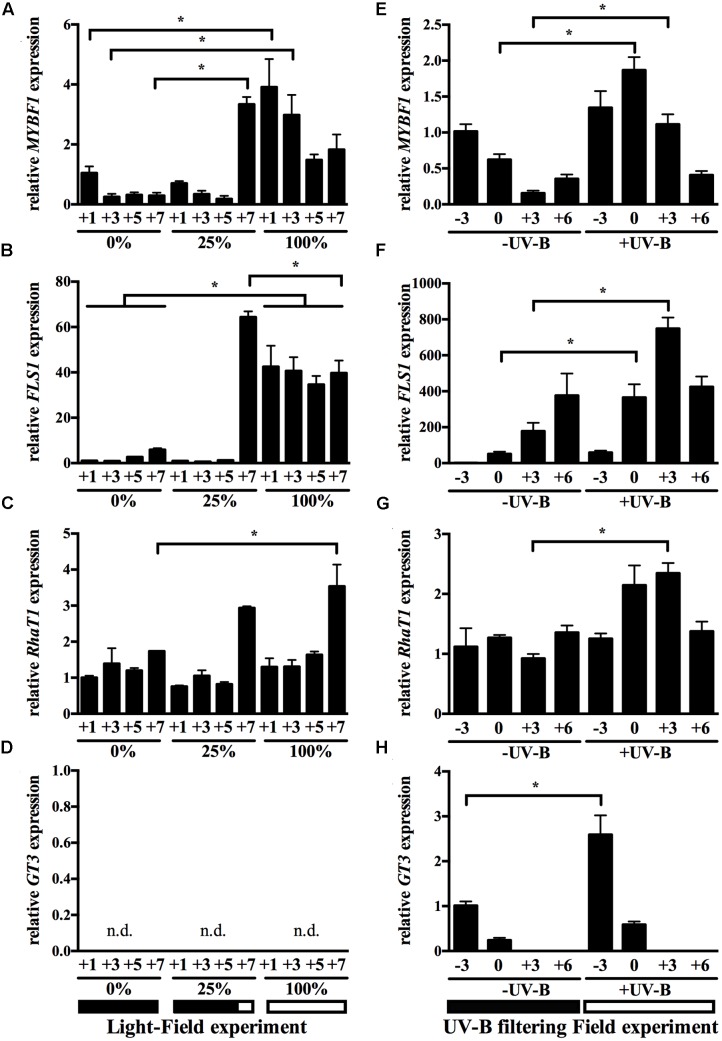
Transcript profiles of known and putative flavonol biosynthetic genes in the berry skin in response to light and UV-B-modifying field conditions. Expression data results for *VviMYBF1* **(A,E)**, *VviFLS1* **(B,F)**, *VviRhaT1* **(C,G),** and *VviGT3* **(D,H)** are shown in two field radiation experiments: cluster sunlight-depletion generated by leaf displacement at field **(A–D)** and UV-B radiation filtering experiment at field (**E–H**, see Materials and Methods). Results from the greenhouse experiments are shown in Supplementary Figure 2. Data points in the UV-B filtering field experiments are given as weeks from the onset of ripening (veraison, time point 0). Sunlight percentages for each treatment refer to the range of time under sunlight exposure: 0% corresponds to full shading of fruits by plant leaves, 100% corresponds to full sunlight exposure from veraison onward, generated by movement of leaves around the cluster region, and 25% shading from veraison until 6 weeks after which leaves were displaced for cluster illumination. Each of the two experiments is exemplified with a diagram at the bottom of the Figure. The experimental design consisted in four blocks with five plants each (biological replicates *n* = 4). Three berries per cluster (randomly sampled) and four clusters per plant were used for each sample. Gene expression in cv. ‘Cabernet Sauvignon’ berries is shown relative to *UBIQUITIN1* expression. Values and standard deviations derived from one PCR run with duplicate PCR reactions on each of the four biological replicates. Stars indicate significant differences at indicated time points across treatments (*p* < 0.05) based on a two-way ANOVA followed by Tukey’s *post hoc* test. Note that the lines in **(B)** indicate that at each time point in 0% versus 100% sun exposure differences in gene expression of *VviFLS1* were statistically significant.

Fruit zone leaf removal is a common viticultural practice that exposes grape berry clusters to sunlight, reducing humidity and improving the accumulation of various secondary metabolites important for fruit quality (Reynolds et al., 1995; Teixeira et al., 2013). In order to evaluate whether gene expression of candidate flavonol-modifying genes is influenced by agronomical practices that alter the exposure of berries to UV-containing light, four experiments were conducted: Experiment 1: sunlight depletion generated by leaf displacement around clusters of field-growing plants (**Figures 5A–D**), Experiments 2–3: control versus ‘High UV-B’ and ‘Low UV-B’ radiation in clusters of 9 year-old plants growing in a greenhouse (Supplementary Figure 2); and Experiment 4: UV-B filtering in clusters of field-growing plants (**Figures 5E–H** and Supplementary Figure 3A). For Experiment 4 not only gene expression levels were measured (**Figures 5E–H**), but also total (Supplementary Figure 3B) and individual flavonol levels determined (Supplementary Figure 4). Furthermore, environmental parameters such as solar UV-B irradiance, temperature, solar radiation at cluster level and total solar radiation were measured (Supplementary Figure 5). For further experimental details for these four light experiments, refer to Section “Light and UV-B Radiation Treatments”.

The MYB TF *VviMYBF1* was strongly upregulated in all developmental stages at 100% exposure in the sunlight field experiment compared with 0% exposure (**Figure 5A**). Its regulation was also higher in the +UV-B condition of the filtering experiment at 0 and +3 WAV (**Figure 5E**). In the greenhouse experiments, *VviMYBF1* expression was more strongly induced by UV-B irradiation (+UV-B) at low intensities but not at high intensities (Supplementary Figures 2A,E). The induction at low intensities was prominent at early stages of development (-3 WAV and time point 0, Supplementary Figure 2B).

Consequently, *VviFLS1*, the target gene of VviMYBF1, was also upregulated by sunlight and UV-B, most prominently at all time points of 100% exposure in the sunlight experiment compared with 0% exposure (**Figure 5B**), and at 0 and +3 WAV in the UV-B filtering field experiment (**Figure 5F**). *VviFLS1* was already induced at -3 WAV, although not statistically significant (**Figure 5F**). Expression of *VviFLS1* was also affected in the greenhouse experiments under UV-B irradiance, especially at +3 WAV and +6 WAV (Supplementary Figures 2B,F).

*VviRhaT1* expression was induced by +UV-B in the UV filtering experiment around veraison (**Figure 5G**) but showed no significant response to 25 or 100% sun exposure in the light field experiment except at +7 WAV (**Figure 5C**) or to low UV-B irradiance conditions in the greenhouse (Supplementary Figure 2G). In contrast to *VviMYBF1* and *VviFLS1*, *VviRhaT1* expression profile throughout development was similar in the 25 and 100% exposure treatments in the sunlight field experiment (**Figure 5C**).

Expression of the three genes *MYBF1* and *FLS1* and *RhaT1* are indicative of a rapid induction to late sunlight exposure (stage +7 WAV in the 25% exposure treatment), as this is the stage when leaves are replaced and clusters are exposed to light (see diagram at the bottom of **Figure 5** and see Materials and Methods) which is supported by a similar expression level of the respective gene at the same stage in the 100% exposure treatment (**Figures 5A–C**).

Regarding *VviGT3*, there is a consistent two-*to*-three-fold induction of its expression in response to UV-B at 3 weeks before veraison in the greenhouse UV-B irradiance or filtering experiments (Supplementary Figures 2D,H and **Figure 5H**). In contrast, *VviGT3* gene expression was not detectable in post-veraison samples such as in the light field experiment (**Figure 5D**), which correlates well with its low expression at late stages of development (**Figure 4E**).

While *VviST1* transcript levels were detectable in the HR lines overexpressing *VviMYBF1*, in none of the light experiments expression of *VviST1* was detectable, also correlating to its low expression found in the cv. ‘Pinot Noir’ developmental series (**Figure 4D**).

In order to correlate gene expression levels with flavonol accumulation, flavonols were quantified in the UV-B filtering field experiment. Flavonols show a significant increase in response to UV-B at different time points (Supplementary Figure 3B). When analyzing for individual flavonol compounds it was found that many flavonols are 3-*O*-glycosylated, as reported before (Carbonell-Bejerano et al., 2014) and the mostly affected were 3-*O*-glucosides of quercetin, kaempferol, and isorhamnetin (Supplementary Figure 4).

## Discussion

### Selection of *VviMYBF1* Transgenic Hairy Root Lines for Microarray Analysis

Several studies have shown that target gene specificity of TFs may differ when introduced into heterologous hosts (Bovy et al., 2002; Luo et al., 2008). Therefore microarray-based transcriptome analysis was performed on the homologous system *V. vinifera* cv. ‘Chardonnay’ HRs overexpressing VviMYBF1 and compared to GFP expressing control roots. For microarray analysis, suitable HR lines were pre-selected by qPCR to detect the presence of significant amounts of *VviMYBF1* and its known target gene, *VviFLS1* (Czemmel et al., 2009). *In situ* staining of transgenic VviMYBF1 HRs for flavonol compounds clearly supported qPCR analysis and demonstrated that flavonols accumulated in VviMYBF1 transgenic roots but not in GFP (control) lines. Flavonol compounds were visible in yellow vacuolar-like cytoplasmic inclusion bodies but not uniformly located in all HR tissues. Interestingly, similar structures named anthocyanin vacuolar inclusions (AVIs) were observed after overexpression of the anthocyanin regulator VviMYBA1 (Cutanda-Perez et al., 2009). The sectors in VviMYBF1 root cross sections accumulating highest levels of flavonols include skin layers (epidermis, hypodermis), endodermis and vascular bundles, whereas no flavonols could be observed in the cortical parenchyma cells. As flavonol regulation by VviMYBF1 is not dependent on the presence of bHLH proteins (Czemmel et al., 2012), a lack of flavonol accumulation in HR tissues such as cortical parenchyma cells might result from either the presence of inhibitors (e.g., transcriptional repressors) or the lack of WD-repeat proteins or other yet unknown activators in these tissues. Main known flavonols found in VviMYBF1 HR extracts were quercetin-3-*O*-glucoside and also quercetin-3-*O*-galactoside. Both of these quercetin-derived compounds have been reported as abundant compounds found in grape leaves and berries (Castillo-Muñoz et al., 2007; Downey and Rochfort, 2008).

### Overexpression of VviMYBF1 in Hairy Roots Identifies Novel Putative Flavonol Pathway Genes Controlled by UV-Containing Light

It is well-known that flavonols, in a similar way as anthocyanins, mainly exist as glycosylated forms whereas corresponding aglycones rarely exist *in planta* (Mattivi et al., 2006). Conjugation with glucose leads to increased water solubility, bioavailability and reduced toxicity, as respective reaction products can be subsequently stored in the vacuole. For example, kaempferol-diglucoside was suggested as one substrate for the tonoplast localized MATE protein Flower Flavonoid Transporter (FFT) in Arabidopsis (Thompson et al., 2010). Sugar moieties at the flavonol core skeleton also influence antioxidant potential (Vinson et al., 1999; Burda and Oleszek, 2001; Montoro et al., 2005) and they are able to protect polyphenols from enzymatic oxidation by plant peroxidases (Regev-Shoshani et al., 2003). These flavonol modifications, also in addition to their content and composition, can be remarkably different among grape varieties, during berry development (Downey et al., 2003; Mattivi et al., 2006; Castillo-Muñoz et al., 2007) and in response to UV-containing light (Price et al., 1995; Czemmel et al., 2009; Matus et al., 2009). Still, major knowledge gaps exist in the regulatory network underlying flavonol biosynthesis under developmental and environmental conditions that determine visible and UV-B light qualities.

Microarray analysis of VviMYBF1 overexpressing HR lines was therefore centered toward the identification of novel structural and regulatory genes involved in flavonol biosynthesis. Promising candidates, namely two glycosyltransferases (*VviGT3*, *VviRhaT1*) and a putative flavonol-sulfotransferase (*VviST1*) were identified using high throughput DE profiling. qPCR analysis of transgenic HRs overexpressing *VviMYBF1* confirmed the microarray results and showed that *VviGT3, VviRhaT1*, and *VviST1* are significantly upregulated (**Figure 4**).

Results in this work clearly support the idea that *VviGT3* is under the control of VviMYBF1 to produce glycosylated flavonols in young developing berries. Despite the many existences of glycosylated flavonols *in planta* and their high importance as sunscreen pigments, when comparing grapevine to the model plant Arabidopsis (Yonekura-Sakakibara et al., 2007, 2008), only a few genes involved in flavonol glycosylation have been characterized *in vitro* (Ono et al., 2010). The same holds true for the identification of only a few structural genes involved in glycosylation of other flavonoids (PAs, anthocyanins) in grapes (Ford et al., 1998; Hugueney et al., 2009; Khater et al., 2012). *VviGT3* is located in the same orientation on chromosome 11 in close vicinity to *VviGT5* and *VviGT6* (Ono et al., 2010). This tandem organization suggests that *VviGT3* arose by tandem duplication and might provide another flavonol modifying gene. Sequence similarity between VviGT3 and VviGT5 (64%) or VviGT6 (64%) is lower compared to the sequence identity between VviGT5 and VviGT6 (88%), which implies functional differences. Although the GT3 protein has not been functionally categorized yet, co-expression analysis reveals some spatial and potential functional differences compared to GT5/GT6. *VviGT5* and *VviGT6* were shown to be co-expressed with *VviFLS1* in berries, leaves and petioles (Ono et al., 2010) with higher *GT5* expressions after the onset of ripening (Loyola et al., 2016). Results from light experiments and berry development show that *VviGT3* transcripts are more profoundly expressed and inducible at early stages of development (**Figure 4E**), which correlates well with peaks of *VviMYBF1* and *VviFLS1* expression in grapes (Downey et al., 2003; Czemmel et al., 2009). Furthermore, *VviGT3* is light inducible only at early stages of development (**Figure 5H**) where it is expressed (**Figure 4E**). The absence of induction in the light field experiment (**Figure 5D**) can be explained by the fact that all samples used there correspond to post-veraison stages where *VviGT3* is low expressed, as it was also demonstrated in the cv. ‘Pinot Noir’ developmental series (**Figure 4E**). *VviGT3* is therefore regulated by light in a similar way as *VviGT5* and *VviGT6* and the flavonol marker genes *VviFLS1* and *VviMYBF1* (Carbonell-Bejerano et al., 2014). Neither in the cv. ‘Corvina’ gene atlas (Fasoli et al., 2012) nor in the cv. ‘Pinot Noir’ series *VviGT3* expression was observed in skin samples of berries toward ripening (**Figure 4E**). These results indicate that at very early stages of development VviMYBF1 could regulate a cascade of *GT* expression profiles including *VviGT3*, *VviGT5* and *VviGT6* to attach sugar moieties [mainly of the class of 3-*O*-galactosides and 3-*O*-glucosides (Supplementary Figure 4)] to flavonol skeletons produced by the co-induced *VviFLS1* gene. Later in berry development, flavonol modifications attached by VviGT3 might not be needed in ripening berry skins, in which the transcriptional network of VviMYBF1 activates the expression of other flavonol structural genes except VviGT3. All UV radiation-increased flavonols found in this study were glycosylated, corroborating the results of Carbonell-Bejerano et al. (2014). The structural similarities between *GT5*, *GT6*, and *GT3* and the pronounced accumulation of 3-*O*-galactosides and 3-*O*-glucosides in the field experiment render the possibility that GT3, besides GT5 and GT6, contribute to the glycosylation of flavonols under UV-light regimes to provide non-toxic UV screens to young berries before veraison.

In conjunction with its albeit modest but present induction by UV-containing light during late stages of development, the *VviRhaT1* gene was induced in *VviMYBF1* HRs when using microarray analysis and confirmative qPCR analysis. The Grape Gene Atlas data showed that cDNA of *VviRhaT1* is present ubiquitously in 50 out of 54 measured samples (Fasoli et al., 2012). It is expressed in all tissues where *VviGT3* is expressed but in addition in many berry and fruit related tissues and additionally in leaves where *VviRhaT1* expression correlates with *VviMYBF1* and *VviFLS1* transcript abundances. This correlates well with the uniform expression of this gene across the cv. ‘Pinot Noir’ developmental series (**Figure 4F**). *VviRhaT1* shows an expression pattern very similar to flavonol accumulation on a per berry basis (Downey et al., 2003). This indicates its involvement in flavonol modification throughout rather than at the beginning of berry ripening at which stage *VviMYBF1* and *VviFLS1* expression peaks (Czemmel et al., 2009). This also supported by a co-induction of quercetin-rhamnoside (+1 WAV) and *VviRhaT1* (0 and +3 WAV) gene expression around or post-veraison in the UV-B filtering field experiment (**Figure 5G** and Supplementary Figure 4H) indicating a putative role of VviRhaT1 as flavonol modifying enzyme. This function is further corroborated by the high sequence similarity to *At2g22590* (*UGT91A1*) from Arabidopsis, which presumably encodes a rhamnosyltransferase under transcriptional control of the flavonol regulators MYB12, MYB11, and MYB111 (Stracke et al., 2007). This regulatory link was identified by transcriptome analysis on a flavonol deficient mutant (*myb11-myb12-myb111*) showing strong downregulation of the transcripts encoding UGT91A1. Transient expression analysis in Arabidopsis protoplasts confirmed that the promoter of *UGT91A1* was responsive to flavonol regulators (Stracke et al., 2007).

Whereas *O*-glycosylation of flavonoids, which may be influenced by VviGT3 and VviRhaT1, has been at least partially studied in grapes, almost nothing is known regarding flavonoid sulfurylation in developing grape berries. In plants, flavonol sulfotransferases (STs) have been initially described in *Flaveria* spp. (Varin and Ibrahim, 1989) and sulfurylated flavonols have often been associated with the nucleus (Grandmaison and Ibrahim, 1995; Naoumkina and Dixon, 2008). In addition, another study suggests that sulfurylated flavonols might play a positive role in the regulation of polar auxin transport by acting as antagonist to quercetin (Faulkner and Rubery, 1992). A detailed examination of grapevine chromosome 11 revealed that genes encoding GTs alternate with two putative flavonol sulfotransferase genes with one of them, *VviST1*, being significantly overexpressed in *VviMYBF1* transgenic root lines but not induced by sunlight or UV-B. Expression data regarding flavonol *STs* in grapes are sparse. The Grape Gene Atlas shows that *VviST1* is only present in 13 out of 54 tissue samples and only in berry pericarp and berry flesh when considering fruit samples (Fasoli et al., 2012). *VviST1* is expressed in roots but not in flowers, which contrasts the expression of *VviMYBF1* in these samples. The low expression, as reported in the Grape Gene Atlas, is supported here when studying expression pattern of *ST1* in developing berries (**Figure 4D**) but thereby shows some correlation with early expression of *VviMYBF1* in developing berries (Czemmel et al., 2009).

Taken together, these results confirm a regulative effect of VviMYBF1 on *VviFLS1*, *VviRhaT1*, and *VviGT3* gene expression in *VviMYBF1* HRs, during berry development and at particular time points in response to sunlight and UV-B radiation. In contrast, greenhouse and UV-B field experiments did not gain any insight on a light-dependent regulation of the putative flavonol sulfotransferase gene *VviST1*. These results extend our knowledge about flavonol glycosylation in response to light and leave open the question whether flavonol sulfurylation can be modified by agronomical practices influencing light.

### VviMYBF1 Is Part of a Regulatory Cascade Involving VviHY5/VviHYH and the PA Regulator VviMYBPA1

Microarray analysis on VviMYBF1 HRs identified the grape *HY5* homolog (VviHYH) and VviMYBPA1 as target of VviMYBF1 (**Table 1** and Supplementary Table 1). While the bZIP factor VviHY5 could not be analyzed in this work because it is not represented on the Grapegen GeneChip, transient expression of VviHY5 in grapevine plantlets resulted in an increase of the expression of *VviMYBF1* (Loyola et al., 2016). Furthermore, the presence of HY5 binding elements typical for bZIP TFs in the promoters of *VviMYBF1* and *VviFLS1* genes was proven by *in silico* approaches (Czemmel et al., 2009). These results suggest a positive feedback loop of VviMYBF1 and VviHYH/VviHY5 in grapevine. A similar relationship is found between the VviMYBF1 and VviHYH homologes AtMYB12 and AtHY5 in Arabidopsis. Lee et al. (2007) identified HY5 binding sites (ACEs) in the promoters of numerous Arabidopsis genes including AtCHS, AtFLS and most prominently AtMYB12 (Lee et al., 2007). This information was used by Stracke et al. (2010) to demonstrate that in Arabidopsis HY5 is required for the transcriptional activation of the AtMYB12 and AtMYB111 genes under UV-B and visible light. This is consistent with the observation that the *hy5* mutant shows flavonoid-deficient roots (Sibout et al., 2006) and downregulation of *AtMYB12* transcripts (Stracke et al., 2010). Taken together, these results support the theory that the UV-B response machinery (including the TFs VviHY5 and VviMYBF1) exist in grapes to propel flavonol accumulation through the activation of the regulatory network consisting of both, MYB and bZIP TFs (Malacarne et al., 2016; Matus, 2016).

Our transcriptome study also demonstrated a downregulation of structural and regulatory genes of the proanthocyanidin (PA) pathway by VviMYBF1 and therefore indicated a competition between the flavonol and PA branch of the pathway (**Table 1**). Indeed, qPCR analysis on VviMYBF1 HRs showed that there is a negative influence of the flavonol regulator VviMYBF1 on the expression of the PA regulatory TF *VviMYBPA1* (**Table 1** and Supplementary Figure 1). Concomitantly, genes which were shown to be under control of VviMYBPA1 such as two *CHALCONE SYNTHASE* isoforms, *ANR* and *CHI* (Terrier et al., 2009) were also downregulated. These results are in line with previous findings that also proposed a competition between flavonol and PA/anthocyanin biosynthesis (Czemmel et al., 2009). Interestingly, an inverse relationship – a differential regulation of *VviMYBF1* expression by VviMYBPA1 – seems not to exist as the expression of the key target of VviMYBF1, *VviFLS1* was not altered in *VviMYBPA1* HRs (Terrier et al., 2009). In context with the contrasting expression profiles of *VviMYBF1* and *VviMYBPA1* in developing cv. ‘Shiraz’ berries (Bogs et al., 2007; Czemmel et al., 2009), the data presented here imply that VviMYBF1 is a positive regulator of flavonol biosynthesis at the expense of PA accumulation via downregulation of *VviMYBPA1* transcription during initial berry ripening stages.

## Conclusion

In this study the grapevine flavonol regulator VviMYBF1 was overexpressed in the homologous model system of cv. ‘Chardonnay’ HRs. Microarrays, qPCR analysis and light/UV exclusion experiments identified promising novel flavonol biosynthetic genes, such as a flavonol glucosyltransferase (VviGT3) and a rhamnosyltransferase (VviRhaT1). These structural genes in context with regulatory genes such as VviHYH, both being under control by VviMYBF1, could play a role in the biosynthesis of flavonols, acting downstream of VviFLS1 and playing a role in UV light responses. Subsequent biochemical characterization of the substrate specificity of the structural candidate genes and the transcriptional potential of the identified VviMYBF1 targeted TFs will foster our understanding of flavonol biodiversity in fruit and thrive the development of molecular tools and agronomical practices to optimize flavonol biosynthesis in response to UV light. As flavonols are more and more explored as important quality determinants of fruit-derived products and have been suggested as quality markers for different grape varieties (Ritchey and Waterhouse, 1999; Hermosín-Gutiérrez et al., 2011), genes involved in flavonol biosynthesis could be implemented as molecular traits during marker-assisted breeding approaches. These techniques will provide tools to the wine industry to optimize fruit quality by adaptation of flavonol content and composition using viticultural practices such as optimization of light regimens.

## Author Contributions

JB conceived and designed the study. SC wrote the manuscript, performed the experiments related to HRs except the qPCR work, analyzed all the data and run the bioinformatics workflow for microarray analysis at QBiC. JH did the qPCR analysis experiments in HRs and developmental series. JM, RL, PA-J, JA performed the sunlight and UV-B greenhouse and field experiments. JM revised the study critically for important intellectual content and together with JH and JB revised the manuscript.

## Conflict of Interest Statement

The authors declare that the research was conducted in the absence of any commercial or financial relationships that could be construed as a potential conflict of interest.

## Footnotes

1 https://portal.qbic.uni-tuebingen.de/portal/
