# Supplementary material for: Transcriptome-Wide Identification of Novel UV-B- and Light Modulated Flavonol Pathway Genes Controlled by VviMYBF1
Source: Front Plant Sci. 2017 Jun 22;8:1084. doi: 10.3389/fpls.2017.01084 (PMC5479930; doi:10.3389/fpls.2017.01084)
Supplement: Supplementary file 2 [file Data_Sheet_1.DOCX]

Supplementary Material

**Transcriptome-wide identification of novel UV-B- and light modulated flavonol pathway genes controlled by VviMYBF1**

**Stefan Czemmel^a,b^, Janine Höll^b^, Rodrigo Loyola^c,d^, Patricio Arce-Johnson^c^, José Antonio Alcalde^d^, José Tomás Matus^e^, Jochen Bogs^a,f,g*^**

**a** Quantitative Biology Center (QBiC), University of Tübingen, Tübingen, Germany

**b** Centre for Organismal Studies Heidelberg (COS Heidelberg), Heidelberg, Germany

**c** Departamento de Genética Molecular y Microbiología, Pontificia Universidad Católica de Chile, Santiago, Chile

**d** Departamento de Fruticultura y Enología, Pontificia Universidad Católica de Chile, Santiago, Chile

**e** Center for Research in Agricultural Genomics (CRAG) CSIC-IRTA-UAB-UB, Barcelona, Spain.

**f** Dienstleistungszentrum Ländlicher Raum (DLR) Rheinpfalz, Breitenweg 71, Viticulture and Enology group, D-67435 Neustadt/W, Germany

**g** Fachhochschule Bingen, Berlinstr. 109, D-55411 Bingen am Rhein, Germany

Correspondence:

Prof. Dr. Jochen Bogs

e-mail: jochen.bogs@dlr.rlp.de

phone +49 (0)6321/671-482

**Supplementary Figures**

**
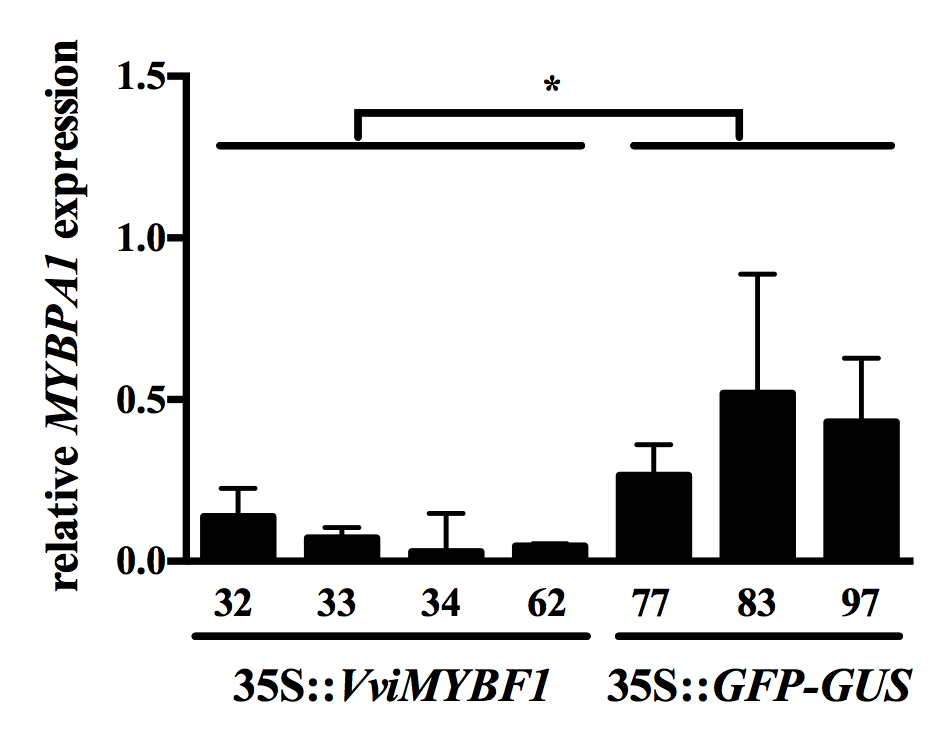
**

**Supplementary Figure 1.** Transcript profile of the MYB TF *VviMYBPA1* in *VviMYBF1* and GFP expressing control hairy roots. Gene expression analysis is shown relative to *UBIQUITIN1* expression. Values and standard deviations are derived from 2 PCR runs with triplicate PCR reactions each. Stars show significant differences (p < 0.05) between MYBF1 and control hairy roots based on T-test.

**
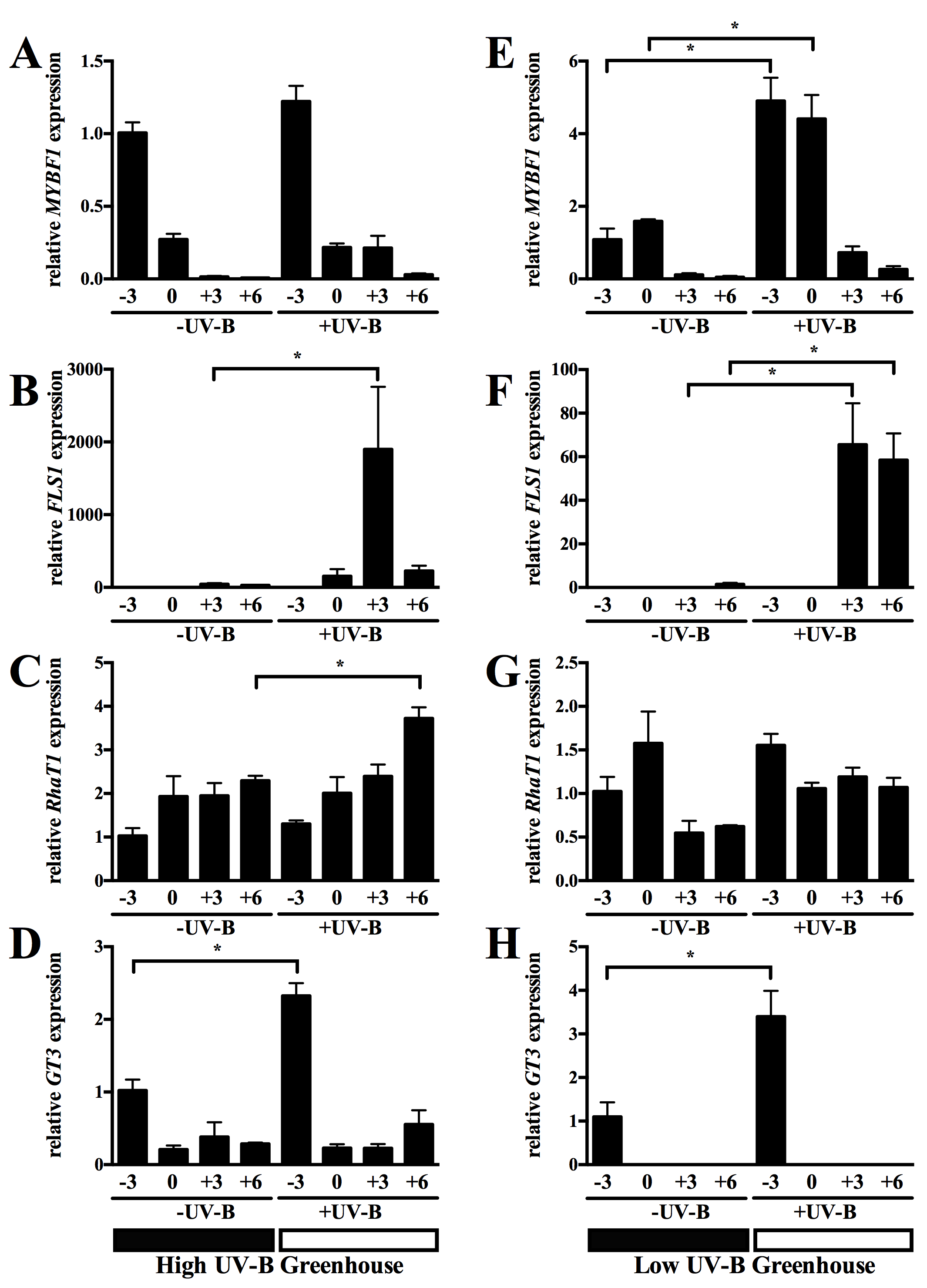
**

**Supplementary Figure 2.** Transcript profiles of known and putative flavonol biosynthetic genes in the berry skin of cv. ‘Cabernet Sauvignon’ in response to high and low UV-B radiation intensities under greenhouse conditions (samples obtained from Loyola et al., 2016). Expression of *VviMYBF1* **(A, E)**, *VviFLS1* **(B, F)**, *VviRhaT1* **(C, G)** and *VviGT3* **(D, H)** was quantified after exposing fruits to two UV-B lamp conditions: i) High UV-B (0.3 Wm^-2^, A-D) and ii) Low UV-B (0.1 Wm^-2^, E-H) irradiation. Data points in the greenhouse experiments are given as weeks from the onset of ripening (veraison, time point 0). Each experiment is exemplified with a diagram at the bottom of the Figure. The experimental design consisted in 4 blocks with 5 plants each (biological replicates n=4). Three berries per cluster (randomly sampled) and four clusters per plant were used for each sample. Gene expression in cv. ‘Cabernet Sauvignon’ berries is shown relative to *UBIQUITIN1* expression. Values and standard deviations derived from 1 PCR run with duplicate PCR reactions on each of the four biological replicates. Stars show significant differences at indicated time points across treatments (p < 0.05) based on a two-way ANOVA followed by Tukey’s post-hoc test.

**
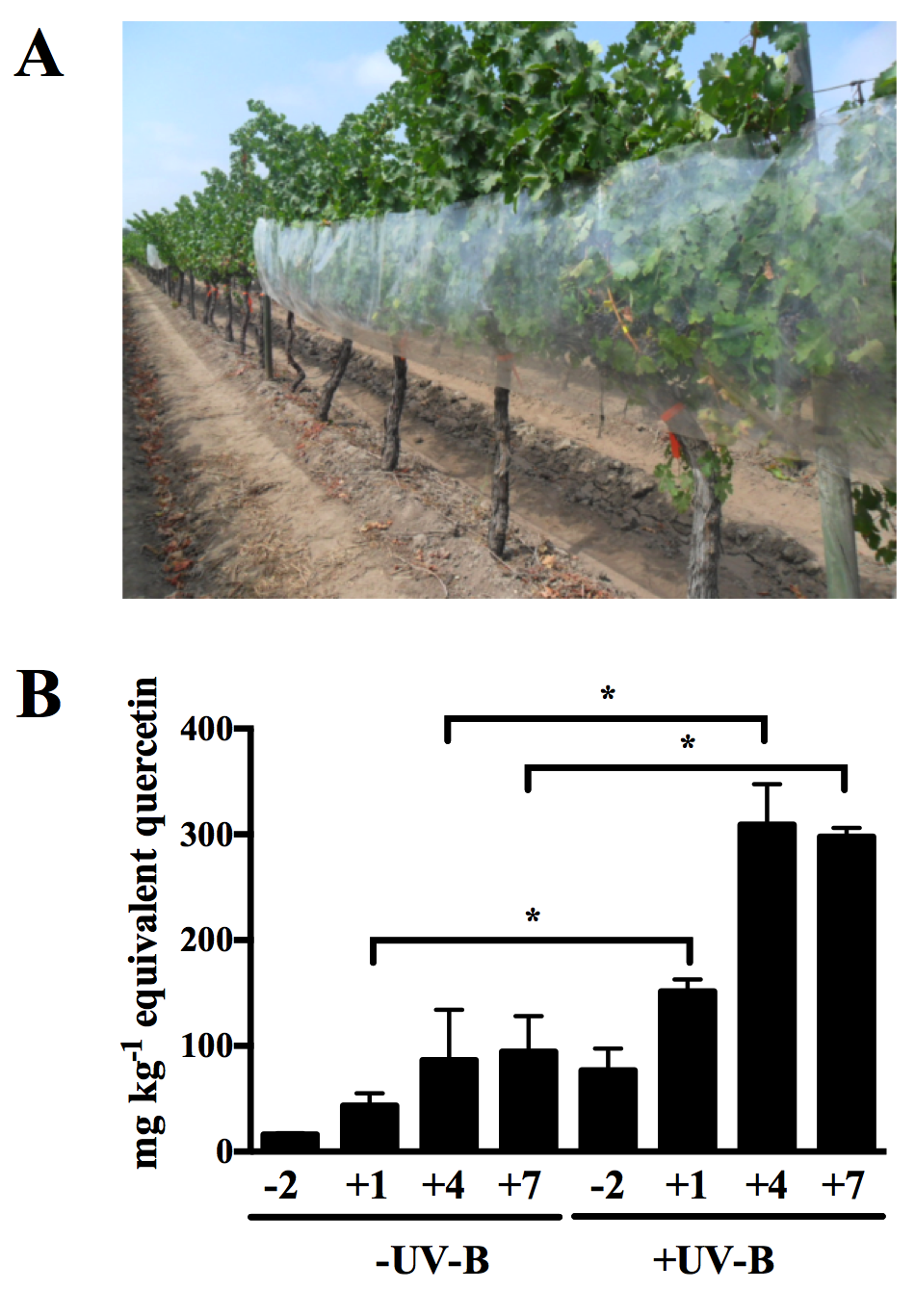
**

**Supplementary Figure 3.** Experimental setup for the field solar UV-B filtering experiment **(A)** and flavonol measurements (**B**). UV-B radiation was filtered using a 100 µm clear polyester film at the grape cluster level (A). Total berry flavonol content (B) was expressed as quercetin-3-O-glucoside equivalents. Berries belonging to grape clusters from the east side of each experimental row were treated and sampled. Values and standard deviations are derived from four independent biological replicates each. Stars indicate significant differences at indicated time points across treatments (p < 0.05) based on a two-way ANOVA followed by Tukey’s post-hoc test.

**
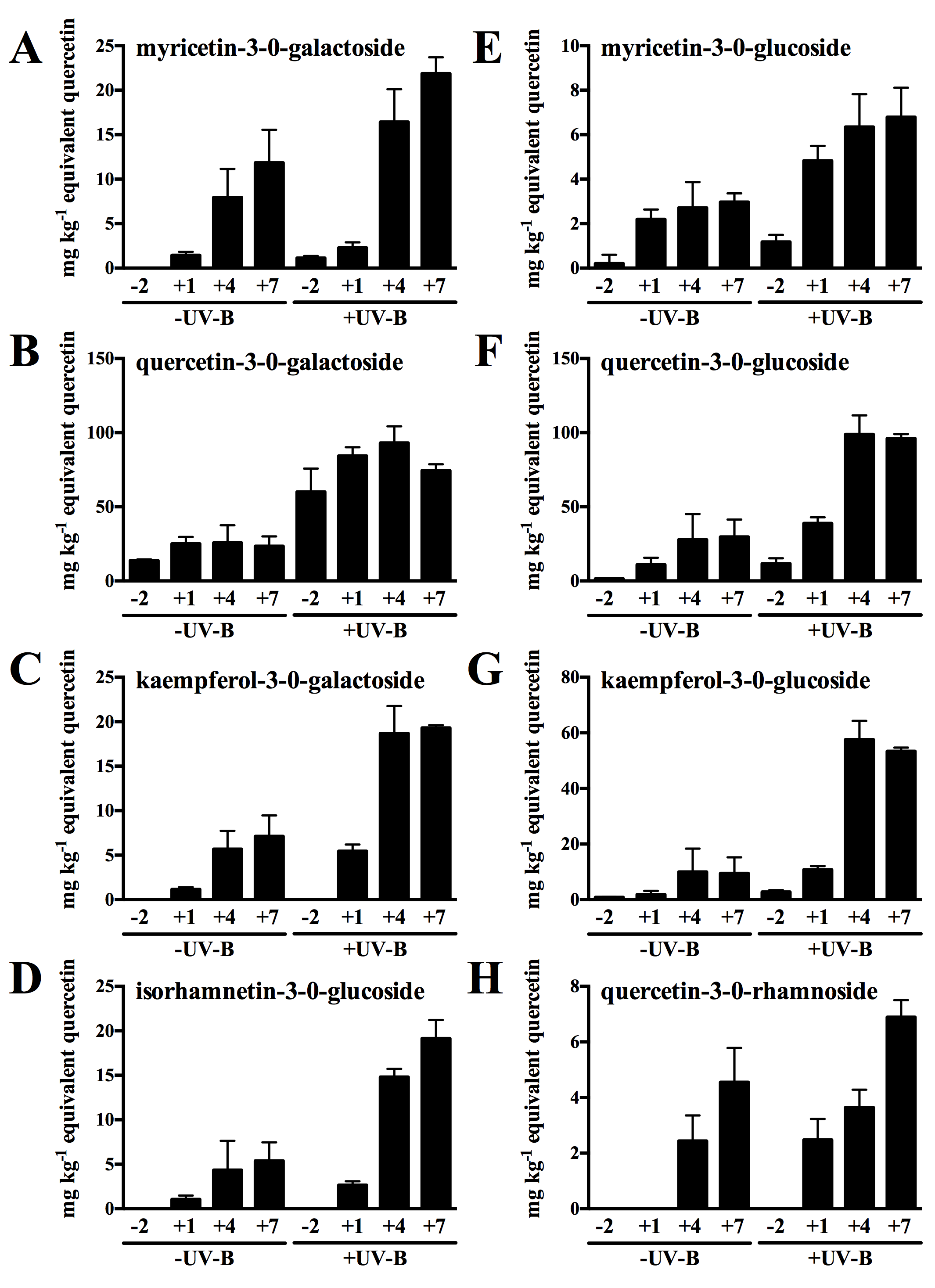
**

**Supplementary Figure 4.** Flavonol-derivative profiles in the berry skin in response to UV-B filtering field conditions. Weeks -2, 1, 4 and 7 after veraison were considered for HPLC quantifications for both -UV-B (filtered) and +UV-B (ambient) treatments. Quercetin was used as calibration standard. Standard deviations (SD) are the result of four independent biological replicates (n=4). No statistical analysis was performed between treatment (+UV-B,-UV-B) groups.

**
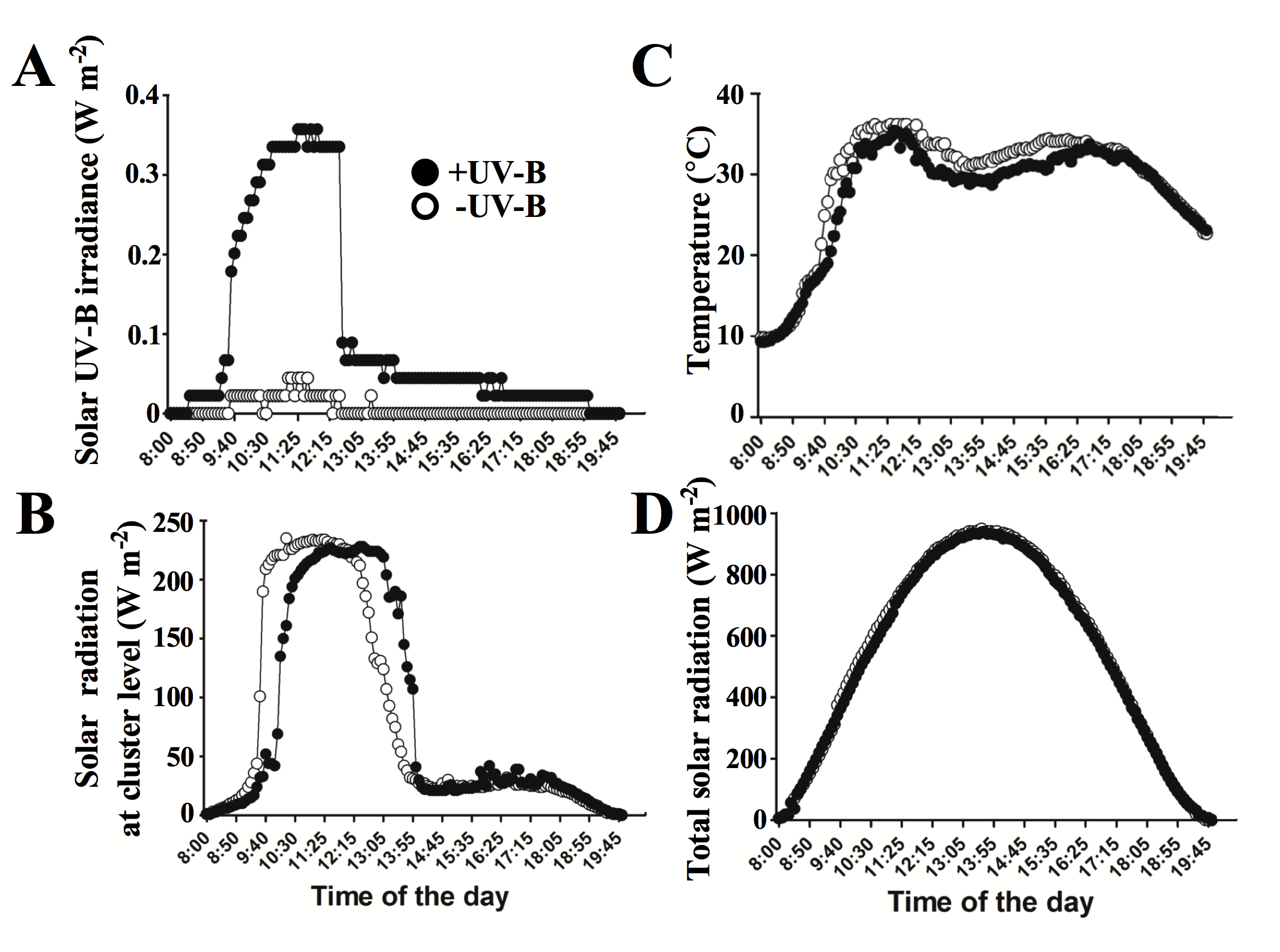
**

**Supplementary Figure 5.** Environmental parameters measured during the UV-B filtering experiment. Solar UV‑B irradiance (A), temperature (B), solar radiation at cluster level (C) and total solar radiation (D) were measured at field conditions. Measurements were taken around veraison from 08.00 h to 19.45 h in the east side of the row.

**Supplementary Tables**

**Supplementary Table 1.** Sheet 1: Complete list of genes from differential expression analysis. Sheet 2: Primers used for qPCR and PCR procedures.
